# A review of methods for the analysis of diagnostic tests performed in sequence

**DOI:** 10.1186/s41512-024-00175-3

**Published:** 2024-09-03

**Authors:** Thomas R. Fanshawe, Brian D. Nicholson, Rafael Perera, Jason L. Oke

**Affiliations:** Nuffield Department of Primary Care Health Sciences, University of Oxford, Woodstock Road, Oxford, OX2 6GG UK

**Keywords:** Sequential diagnostic testing, Diagnostic accuracy, Conditional dependence, Diagnosis

## Abstract

**Background:**

Many clinical pathways for the diagnosis of disease are based on diagnostic tests that are performed in sequence. The performance of the full diagnostic sequence is dictated by the diagnostic performance of each test in the sequence as well as the conditional dependence between them, given true disease status. Resulting estimates of performance, such as the sensitivity and specificity of the test sequence, are key parameters in health-economic evaluations. We conducted a methodological review of statistical methods for assessing the performance of diagnostic tests performed in sequence, with the aim of guiding data analysts towards classes of methods that may be suitable given the design and objectives of the testing sequence.

**Methods:**

We searched PubMed, Scopus and Web of Science for relevant papers describing methodology for analysing sequences of diagnostic tests. Papers were classified by the characteristics of the method used, and these were used to group methods into themes. We illustrate some of the methods using data from a cohort study of repeat faecal immunochemical testing for colorectal cancer in symptomatic patients, to highlight the importance of allowing for conditional dependence in test sequences and adjustment for an imperfect reference standard.

**Results:**

Five overall themes were identified, detailing methods for combining multiple tests in sequence, estimating conditional dependence, analysing sequences of diagnostic tests used for risk assessment, analysing test sequences in conjunction with an imperfect or incomplete reference standard, and meta-analysis of test sequences.

**Conclusions:**

This methodological review can be used to help researchers identify suitable analytic methods for studies that use diagnostic tests performed in sequence.

**Supplementary Information:**

The online version contains supplementary material available at 10.1186/s41512-024-00175-3.

## Background

Statistical methods for summarising the results of single diagnostic tests are well established, with guidelines for performing and reporting diagnostic studies available [1, 2]. Clinical diagnostic pathways consist of a number of investigatory tests or procedures with the aim of determining a diagnosis. In practice, most such pathways require more than one diagnostic test to be performed before a diagnosis can be made [3]. For example, laboratory testing to identify patients at highest risk of cancer may be followed by imaging to visualise likely cancer, then biopsy to provide a tissue diagnosis of suspicious lesions. A review found that 16 out of the 22 diagnostic pathways in diagnostic Health Technology Assessments published between 2009 and 2015 contained multiple diagnostic tests [4].

If the diagnostic pathway is considered fixed and known in advance, and a diagnostic accuracy study can be performed to test the pathway in its entirety, the analytical steps required may be similar to those for a single diagnostic test. However, in practice model development is often required to develop a strategy for assigning positive and negative diagnoses within a pathway, and the resources needed to conduct a study of the full pathway may be prohibitive. Often, only partial information about each of the component tests is available, sometimes from different studies conducted in different settings. In this scenario, constructing and assessing the full diagnostic pathway requires the dependence between the results of the tests to be incorporated, using either additional assumptions or published estimates [5].

In some studies, several different diagnostic tests are performed at the same time, and the results may be combined to reach a diagnostic decision. These may presented in the form of a clinical risk score, such as when using blood test results in combination to assess cancer risk [6]. Previous methodological papers have suggested ways for creating optimal diagnostic combinations, using a variety of methods including forms of regression, discrimination analysis and methods based around maximising the area underneath the Receiver Operating Characteristic (ROC) curve [7–14].

**Fig. 1:**
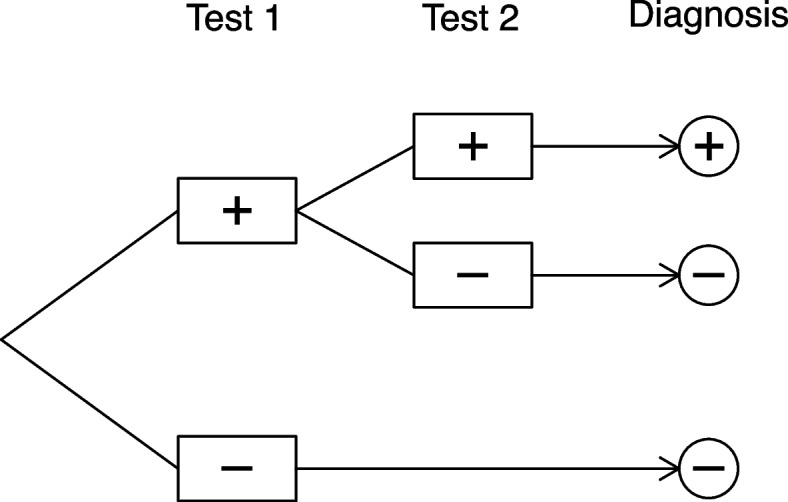
Illustration of conditional diagnostic test sequence

In other scenarios, diagnostic tests are performed in sequence, separated in time, such that the result of one test is known before subsequent tests are performed. An example is in repeat point-of-care testing for SARS-CoV-2 [15]. Another example is in developing criteria for diagnosing latent tuberculosis in which two tests are available (tuberculin skin test and an interferon gamma release assay); one strategy is to offer the skin test first, then perform the array only for individuals who have a positive result from the skin test [16]. In such cases, the decision to perform diagnostic tests that fall later in the sequence depends on the results of those already observed. Typically, the diagnostic decision would be based on the final test performed in the sequence, but whether the final test is conducted will depend on the results of the preceding tests (Fig. 1).

This pattern of tests being performed in sequence is known as ‘conditional testing’ or ‘serial testing’, and contrasts with ‘parallel testing’, when all tests are performed on all participants irrespective of their results [17, 18]. In Fig. 1, if Test 1 gives a negative test result, an outright negative diagnosis is made without Test 2 being performed, which may be desirable in terms of minimising the burden of testing on the patient and reducing costs. However, a false negative result from a Test 1 that had poor sensitivity for the target condition would prevent Test 2 being used in patients with the target condition, and this would adversely affect the performance of the sequence even if Test 2 had high sensitivity. This conditional design, which has previously also been termed the ‘paired’ diagnostic study design [19], is easily extended to encompass additional tests or more complicated decision rules.

Although many methods for analysing diagnostic test sequences have been proposed, a review of these methods is lacking. The objective of this paper is to provide such a review, where the aim is generally to estimate the overall diagnostic performance of the test sequence. The majority of the paper consists of an overview of available methods, grouped into themes. An analysis of a dataset consisting of a diagnostic test sequence for colorectal cancer illustrates some of the methodological considerations. A concluding discussion summarises our findings and describes future research priorities.

## Methods

We followed a three-stage process for identifying papers for inclusion in this review. In the first stage, we identified 18 papers that were known to us and thought relevant to the topic of interest. In the second stage, we performed a literature search using the PubMed database, with the search restricted to five journals that had previously published papers in this area and which we considered to be likely sources of additional eligible publications: *Biometrics*, *Biostatistics*, *Journal of Biopharmaceutical Statistics*, *Statistical Methods in Medical Research* and *Statistics in Medicine*.

In the third stage, we took all eligible papers from the first two stages and used Scopus and Web of Science to perform a one generation forwards and backwards citation search (i.e. extracting their reference lists, and all publications that had subsequently cited papers identified in the first and second stage) [20]. In this final stage there was no restriction on journal.

At each stage, papers were screened independently by two researchers for appropriateness, first by title/abstract, then using the full text if required. Included papers are those that describe statistical methods for assessing the diagnostic accuracy (i.e. the diagnostic performance, typically measured by parameters representing the sensitivity, specificity or predictive values) of any of the following:

- Two or more distinct tests performed in sequence or as part of a pathway
- A series of repeated tests, i.e. the same test performed on two or more occasions in sequence
- Test sequences carried out as part of a screening programme

Papers that incorporate issues relating both to sequential testing and other important methodological issues, such as allowing for an imperfect or partial reference standard, were also included.

The following were excluded:

- Primary studies that evaluate particular diagnostic tests
- Systematic reviews of particular diagnostic tests
- Methods for sequential hypothesis tests used for purposes other than diagnostic accuracy evaluation
- Papers that solely aim to estimate prevalence rather than evaluate diagnostic accuracy
- Methods for comparing the performance of two or more diagnostic tests against each other with the aim of determining which test has superior performance (discussed elsewhere [21]) rather than incorporating them into a single pathway

Given the difficulty in searching for suitable papers in this field, the search strategy was not designed to be systematic but rather to enable us to identify the most prominent classes of methods that have been proposed for analysing sequences of diagnostic tests, for the purpose of creating a methodological overview. From eligible papers, we extracted information about the objective of the analysis and the type of statistical method described, and used this information to group papers sharing similar methodology into themes. We illustrate one of the key methodological issues - the potential effects of conditional dependence between tests - with an example that considers repeat use of the Faecal Immunochemical Test for colorectal cancer in symptomatic patients. The primary focus of the paper is on cases where the index test results and the true disease status are binary, as this is the scenario that has arisen the most often in the clinical settings in which these methods have been applied, but we also refer to methods for continuous index tests where appropriate.

## Results

This section contains the five groups of methodologies that we created following the literature review. Table 1 summarises the key features of these groups. A further overview that can guide users to the most appropriate subsection of the results for a particular study design or objective appears as Supplementary Material.

**Table 1:** Summary of analytical considerations in each group of methodologies

Section	Examples of applicable situations
1. Combining the results of index tests performed in sequence	• Incorporate the results of two or more tests to create an overall diagnostic decision
	• Assess the effect of adding a new index test to a test sequence
	• Most suitable if all index tests are performed on all participants
2. Estimating conditional dependence between index tests performed in sequence, and conditional testing	• Assess the conditional correlation between index test results
	• Suitable when there is conditional testing, with the decision to perform later tests in the sequence dependent on earlier test results
	• Suitable if some index tests are not performed on all participants
3. Analysing test sequences used for risk assessment	• Assess the performance of a repeating sequence of the same index test
	• Typically arises in the context of diagnostic testing for screening or monitoring
4. Analysing test sequences in conjunction with an imperfect or incomplete reference standard	• Adjust the performance of a test sequence for a reference standard that is imperfect
	• Assess a test sequence if the reference standard is missing or performed on only some participants
5. Meta-analysis	• Synthesise test sequence performance data from multiple studies

### Combining the results of index tests performed in sequence

#### Combining two index tests

##### The AND and OR rules

In the simplest case, two binary index tests are performed on all participants, yielding results $X_1$ and $X_2$ respectively. We use *T* to denote true disease status. Two simple combination rules are possible. The first is the ‘OR’ rule, also known as ‘believe the positive’ or ‘Any+’, in which a positive diagnosis is made if either $X_1$ or $X_2$ is positive. The second is the ‘AND’ rule, also known as ‘believe the negative’ or ‘Both+’. In these cases, $X_2$ can also be viewed as an ‘add-on’ test to an established test $X_1$, or $X_1$ as a new triage test to an established test $X_2$ [22]. In a third rule, ‘believe the extreme’, $X_2$ is performed only among participants who have an indeterminate result for $X_1$, and a positive diagnosis is made if either result is positive [23].

Under these rules, algebraic expressions are available for the sensitivity and specificity of the combination in terms of the sensitivity and specificity of the individual tests and the conditional dependence between them. These are shown in the Appendix. The presence of conditional terms such as $\text {P}(X_2|X_1,T)$ in these expressions highlights the direct influence of conditional dependence between the index tests on the diagnostic performance of the sequence.

##### Understanding the effects of conditional dependence

Estimating conditional probability terms that include both $X_1$ and $X_2$ can be problematic if data are available only from studies that have evaluated the diagnostic performance of each of the tests in isolation. The simplest approach is to assume that $\text {P}(X_2=0|X_1=0,T=1)=\text {P}(X_2=0|T=1)$, making the strong assumption that $X_1$ and $X_2$ are conditionally independent given disease status [24]. More generally, it can be shown that the combined sensitivity and specificity can be re-expressed in terms of the measure known as the phi coefficient (or conditional correlations $\rho _{+}$ and $\rho _{-}$ between the index test results, given positive or negative true disease status) for measuring association between binary variables (see Appendix) [25].

The possible range of the conditional covariance between $X_1$ and $X_2$ is constrained by the marginal sensitivity and specificity of each test within the sequence [26]. In an extreme case, if $X_1$ and $X_2$ are conditionally perfectly negatively correlated, they would always give discordant results. In this case, the sensitivities of the OR and AND rules would become one and zero respectively, while their specificities would become zero and one respectively.

Under the OR rule, the combined sensitivity is increased when the two index tests are conditionally negatively correlated, given disease positivity (see formula (1), Appendix). Analogously, the combined specificity under the OR rule is increased when the two index tests are conditionally positively correlated, given disease negativity [27, 28]. The opposite conclusions apply under the AND rule [29]. The relationship between the strength of the conditional correlations and the overall performance of OR and AND rules has been explored numerically [30].

The effects of the OR and AND rules on positive and negative predictive values have also been examined. In most realistic scenarios, the OR rule tends to decrease the PPV and increase the NPV, and the AND rule tends to increase the PPV and decrease the NPV, although this tendency is not guaranteed and may not occur if the index tests are conditionally highly correlated, with differential strength of correlation among disease-positive and disease-negative cases [31]. These effects have also been demonstrated in experimental examples [32].

##### Other measures of diagnostic performance

The additional benefit of adding a new index test to an existing index test or test sequence can also be quantified using positive and negative likelihood ratios, allowing the incremental gain in performance to be assessed [33], by tests based on the difference or proportion of additional correct diagnoses [34], or by measures such as the relative true or false positive rates and relative ROC curve [35]. The same principle can be extended from evaluation of diagnostic accuracy to the evaluation of cost in health-economic terms [36], if the aim is to minimise the overall cost associated with the test sequence and the relative costs of correct/incorrect positive/negative diagnoses can be quantified [23, 37]. This can be used to test whether the diagnostic benefit of implementing subsequent tests, based on OR or AND decision rules, justifies the additional cost [38].

Some authors have examined the effect of adding an index test on the weighted kappa statistic for agreement between test results, although this measure can be difficult to interpret as a measure of clinical performance [18, 39]. An alternative method, which aims to control false positive and/or false negative error rates within prescribed limits, stochastically designates a proportion of those with discordant index test results as positive and the remainder as negative, although this has the disadvantage that two individuals with identical test results may as a consequence be assigned different diagnoses [40].

Expressions for the combined sensitivity and specificity of a test sequence when either index test is continuous, with test positivity defined based on exceeding a specified threshold, are available elsewhere [41]. Thresholds can be either fixed or adaptive - changing over time, depending on the ordering of the test sequence. The latter scenario is particularly relevant to the analysis of diagnostic test sequences, because the expressions for sensitivity above show that the sensitivity of the overall test sequence can be expressed in terms of the sensitivity of the first test result and the conditional sensitivity of the second test result given the first. If the unconditional and conditional diagnostic performance parameters of all tests in the sequence are known, an optimisation search can be performed to identify the thresholds that achieve a specified target diagnostic performance of the full sequence [41].

#### Combining more than two index tests

These ideas can be extended to situations in which more than two index tests are available. Even when test results are binary, the number of possible test combinations and test sequences increases exponentially with the number of tests, and most of these combinations cannot be expressed as simple OR or AND rules. Some named general rules suitable for more than two tests include the ‘majority’ rule (in which a positive diagnosis is made if more than half of the individual test results in the sequence are positive) and the ‘unanimity’ rule (a generalisation of the AND rule in which a positive diagnosis is made only if every test result in the sequence is positive) [39, 42]. If there are many index tests, selecting an optimal combination can be viewed as an optimisation problem akin to the combinatorial ‘knapsack problem’, well-known in operations research [43], although this fails to allow for sampling variability in the index tests [44, 45].

If suitable data are available for modelling, methods for combining multiple tests can be generalised to continuous time, for example by monitoring whether a longitudinally-modelled biomarker exceeds a (possibly time-varying) threshold for disease positivity at any point during follow-up [46].

Some methods assume that a mechanism for defining the diagnostic decision from a sequence of index tests has been pre-defined. These methods often also assume that the performance of the individual index tests, and the conditional correlation between them, are known. This can inform decisions about, for example, the choice of the number of repeated screening tests [47], or the optimal ordering of tests to minimise costs while controlling specified rates of misdiagnosis [42]. With similar objectives, a ‘probability-modifying plot’ has been proposed to illustrate graphically how the conditional probability of disease changes after index test results are added sequentially [48].

##### Creating a new rule for combining results from a test sequence

In the situation when a mechanism for defining the diagnostic decision from a sequence of index tests has not been pre-defined, existing multiple variable analysis methods have been adopted. These methods include logistic regression (which is suitable for both binary and continuous index tests), discriminant analysis and ‘distribution-free’ methods that are based on maximising quantities such as the Mann-Whitney U-statistic estimator of the area under the ROC curve [49, 50]. In their usual form, these methods are generally only applicable if results are available for index tests on all participants.

### Estimating conditional dependence between index tests performed in sequence, and conditional testing

In contrast with the previous section, scenarios often arise in which not all diagnostic tests forming the test sequence are performed on all participants. Typically, the decision to perform subsequent tests may depend on the test results seen so far (‘conditional testing’). In these scenarios, estimating the dependence between index tests results, conditional on true disease status, becomes more important, as this affects the diagnostic performance of the sequence as a whole. This section outlines general principles that are illustrated in the later case study.

#### Formulating a test sequence with known parameters

Hershey et al. were among the first to consider a mathematical formulation of diagnostic test sequences and their effect on clinical utility (i.e. a quantifiable impact on health outcomes) [17, 51]. They did this by deriving algebraic expressions for the clinical utility of test sequences allowing for the possibility of conditional testing, and considering changes in the ordering of tests within the sequence. This is the scenario illustrated in Fig. 1. These expressions allow the utility of different diagnostic testing strategies to be compared. Their approach assumes that all relevant parameters are already known - not only the disease prevalence and the diagnostic performance estimates, but also the conditional dependence between test results and measures of clinical utility. Their results suggest that these parameters in combination dictate the optimal diagnostic test sequence, and that this is context-specific, to the extent that for some parameter combinations, additional diagnostic testing may be detrimental to the overall performance of the sequence [51].

Levy and Kass derived maximum likelihood estimators for disease prevalence and test specificity, assuming known test sensitivity, in a conditional testing sequence with three stages, with further testing performed only on those who tested positive at a given stage, in the context of screening for bacteriuria [52].

#### Parameter estimation in the presence of conditional testing

Estimators of diagnostic performance that are derived from a study that uses conditional testing, but do not account for the conditional testing, are in general biased, with the size of the bias depending on several factors, including the disease prevalence and the conditional dependence between the index test results. This has been demonstrated both theoretically [27, 53] and empirically through simulation studies [54].

For this reason, methods have been developed to test for the presence of conditional dependence between index tests within a test sequence. One such method for binary test results is a likelihood-based test of the hypothesis that the conditional correlation parameter $\rho _{+}=0$ (and/or $\rho _{-}=0$) [55]. The level of conditional dependence between different index tests has been proposed as a way of deciding an ordering of them as a test sequence through the repeated use of Bayes’s theorem [56]. This idea has been developed further using Bayesian network methodology to model the interdependence between several index tests and a reference standard [57].

One approach to modelling data from a test sequence while allowing for conditional dependence between test results models the data as a realisation of a multinomial distribution whose parameters are determined by the outcome prevalence $\pi$ and the sensitivities and specificities of the test sequence (see Appendix for expression of likelihood function) [58]. In some cases it may be possible to estimate the required conditional probability terms from the available data, but this model can also be used when reference standard information is not available or is incomplete, so is described further in the section Analysing test sequences in conjunction with an imperfect or incomplete reference standard.

The conditional dependence can also be conceptualised by regarding a binary test result as being derived from a measure exceeding a particular positivity value *c* on an underlying, or latent, continuous trait scale *U*. Here the conditional dependence is governed by the correlation parameters on this latent scale [59]. In this case, the expressions $\text {Cov}(X_1,X_2|U \ge c)$ and $\text {Cov}(X_1,X_2|U < c)$ provide the conditional covariances between the index tests, given the true diagnosis and the positivity threshold, which can in turn be used to derived the analogues of the conditional correlations $\rho _{+}$ and $\rho _{-}$. Assuming a distributional form for *U*, with a particular measurement error variance, induces a conditional correlation between the index test results even if these measurement errors are themselves independent [59].

These ideas can be extended to two latent variables, $U_1$ and $U_2$, linked to $X_1$ and $X_2$ respectively, by inducing a correlation between $X_1$ and $X_2$ via specifying a copula function - a joint distribution function whose univariate marginal distribution functions are the distribution functions of $U_1$ and $U_2$ [60]. In most scenarios, this model is not identifiable, and so parameters must either be specified in advance or assigned informative priors, and the copula approach has not gained widespread adoption ahead of other latent variable methods or methods in which dependence between index tests is specified or estimated directly.

### Sequences of diagnostic tests used for risk assessment

A particular form of conditional sequential testing may arise when assessing the risk of future ill health. For example, such a sequence may occur when adults are routinely screened for raised blood pressure, or when patients with diabetes are monitored to assess their risk of developing sight-threatening retinopathy.

Although conceptually similar to sequential testing for other purposes, test sequences for risk assessment may differ in at least two respects. Firstly, the criteria for progression may differ in that high sensitivity during early phases is often desirable [61]. Secondly, many screening and monitoring strategies allow for the possibility of repeated testing using the same diagnostic test, whether as part of a conditional sequence of tests or because check-ups are routinely performed at intervals over time [62]. The testing patterns used in risk assessment may therefore differ from those used in other diagnostic settings, and consequently alternative methods have been developed with these considerations in mind.

#### Developing a diagnostic strategy from multiple repeated test results

**Fig. 2:**
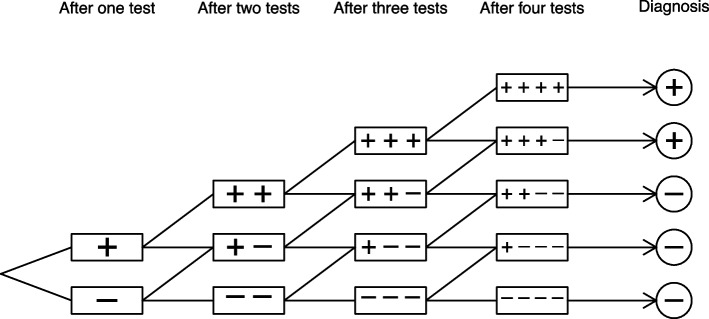
Illustration of screening sequence with $n=4$ and $k=3$. In this example, it is assumed that the ordering of test results within each diagnostic sequence does not affect the diagnostic decision

The particular scenario in which all individuals are screened on *n* occasions, and a screen-positive result is declared if they test positive on at least *k* occasions, has been examined in two related papers by Lau [63, 64], following much earlier work by Nissen-Meyer [65]. In these papers, it is assumed that estimates of diagnostic performance of a single test are known, and the objective is to determine values of *n* and *k* such that the performance of the test sequence meets a given criterion, such as a particular false positive and false negative detection rate, as shown in Fig. 2.

This procedure is first developed for sequences in which test results in an individual are considered conditionally independent [63], and then, via canonical moments, for sequences that allow for dependence between results [64]. Similarly, methods for estimating the probability that a false positive diagnostic test result will occur at some stage within a sequence of test results have been developed both assuming non-conditional testing [62] and assuming conditional testing [66]. Sequences in which participants receive further tests only if they have received positive results at the previous stage can be modelled using conditional multinomial distributions [67], and this approach has been extended to allow for heterogeneity across population subgroups using participant-level covariates [68].

If the index test is based on a continuous measurement, the shape of the ROC curve of the test sequence can be modelled as a function of the between-test correlation and the number of tests performed per participant, which may be conditional on the test result. The apparent diagnostic performance of the sequence is affected if these factors are not accounted for [69].

#### Decision-theoretic methods

Decision-theoretic methods, based on cost criteria, have been developed for the optimal interpretation of the results of a screening sequence in the case $n=2$ using a Bayesian framework [70], and for optimising the choice of *k* [71]. These methods are perhaps more suitable for sequences in which the same test is performed on more than one occasion, but they can also be applied to sequences in which different index tests are used within the sequence, provided the diagnostic performance of each test is known.

More general questions of how a risk assessment programme should be organised, considering factors such as the optimal scheduling of tests, or replacing one screening test with another, are beyond the scope of this article but have been examined from a methodological perspective elsewhere [72, 73].

### Analysing test sequences in conjunction with an imperfect or incomplete reference standard

A common problem in the analysis of sequences of diagnostic tests, especially those that incorporate conditional testing, is misclassification of test results by the reference standard. This may arise either if all subjects receive the reference standard but some are diagnosed incorrectly (usually termed an ‘imperfect’ reference standard), or if some or all subjects do not undergo the reference standard test. The last of these situations is often called ‘partial verification’.

**Fig. 3:**
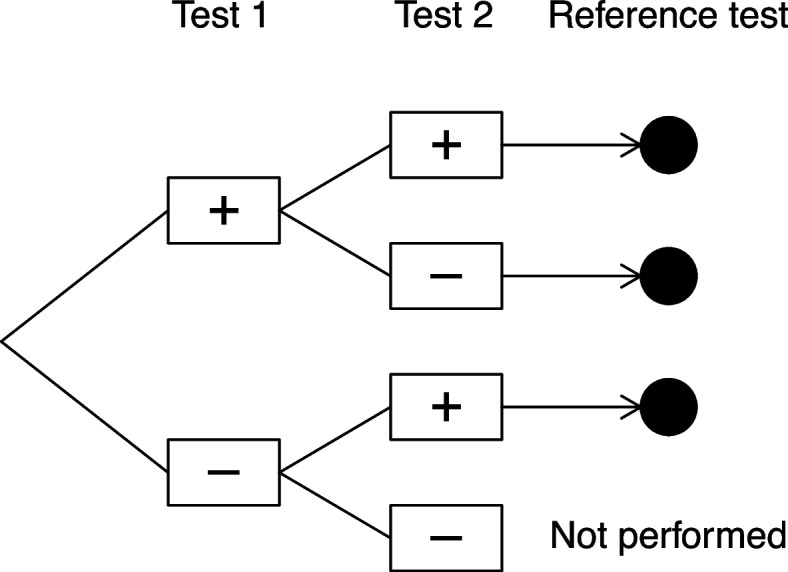
Illustration of screening sequence that is typical of partial verification. In this case, only individuals who test positive on one of the two index tests receive the reference standard test

This topic is not specific to evaluating test sequences, and reviews of methods to allow for imperfect reference standards [74], missing reference standards [75] and partial verification [76] have been published previously. Nevertheless, it often arises in sequential test scenarios and the use of a reference standard is sometimes itself conditional on index test results, for example in screening programmes in which individuals testing positive initially are more likely to undergo subsequent confirmatory testing than those whose initial test result was negative (Fig. 3) [77–79]. Unadjusted estimates of diagnostic accuracy are biased when there is incomplete disease status ascertainment [53], and adjusted maximum likelihood estimators have been derived [80]. Methods relating to the role of imperfect or partial verification that are likely to be of most use in analysing test sequences are summarised here.

#### The multinomial latent variable model for reference standard adjustment

Some methods that attempt to allow for both between-test dependence and an imperfect or missing reference standard are based on the multinomial model. This model can be parameterised in various ways to capture the between-test correlation. One suggested parameterisation arises from the observation that joint probabilities of test results, such as the expression for the sensitivity of the AND rule, can be re-expressed using conditional covariance parameters (see Appendix for equation). The values of these covariance parameters are constrained within a particular range given the diagnostic performance of each index test, and this can be used to obtain upper and lower bounds for diagnostic accuracy estimates if there is an imperfect reference standard [28, 81].

This parameterisation was introduced by Vacek [28] and has been used frequently since [26, 58, 82–85]. In a Bayesian framework, the choice of parameterisation can be guided by the ability to specify plausible priors for the parameters. This is important as the fully-parameterised model is not identifiable, and so some of the parameters require either fixing deterministically or being assigned an informative prior [58]. The model has been extended to allow individual-level covariates to be incorporated [82, 83] and adapted for the specific scenario of multistage diagnosis to allow combined diagnostic criteria to be compared with those based on a single test [86]. The approach of Vacek, which is suitable for two index tests, has been generalised to allow for multiple (three or more) index tests [85]. If multiple index tests are separated in time, as might often occur in sequential diagnostic testing, the covariance between them can additionally be modelled as a function of the time between them [87].

An alternative formulation allows the conditional dependence between test results to be modelled in terms of individual-level random effects, which have been interpreted as a latent measure of disease ‘intensity’ [26, 88]. The individual-level random effect induces a correlation between tests performed on the same individual without needing to specify separate covariance parameters. Again, inference will often require the specification of informative priors. A comparative study of several latent variable methods for modelling the joint dependence between index tests has been performed [89].

#### Latent class models for a missing reference standard

Latent class models may be attractive in situations when the reference standard is entirely missing. In this case, the latent variable *U* is a measure of true disease status, which may also be modelled on the continuous scale (for example, as an unobserved $\text {N}(0,1)$ variable), and diagnostic accuracy parameters are expressed conditional on *U*. With suitable constraints on parameters, estimation may use either the EM algorithm [90] or a Bayesian framework [91]. In its basic form, this model does not account for the ordering or timing of the test results that might occur as part of a test sequence; one way of extending it is to replace *U* with a latent process that evolves over time, such as via a Markov process [92]. Latent variable methods have been described both assuming conditional independence between index tests [93] and more generally, relaxing this assumption [94].

Because of identifiability concerns, latent variable models may be especially useful in cases where there are many index test results, including cases when there is multiple rater assessment using a large number of raters [95, 96]. One constraint has been described as a ‘rule of three’, by which identifiability is assured in the sequential testing case provided at least three index test results are observed on each participant [97], and more general identifiability issues are discussed in detail elsewhere [98].

Although latent variable models have been used most often in the diagnostic test literature for binary index test results, a similar model can be used if index tests give a continuous measurement [99]. Continuous index tests can also be modelled as arising from a mixture distribution, governed by separate parameters for disease-positive and disease-negative individuals, even if the true disease status is unknown (under weak assumptions), and the results of this model have been used to try to construct an optimal sequence of index tests [100].

#### Partial verification

Various methods have been proposed to analyse test sequences in conjunction with partial verification, dictated by the nature of the data available. The common challenge that underpins these methods is the non-identifiability of standard models that is caused by incomplete reference standard data. For example, in a study in which disease verification is only available for participants who test positive for either of two index tests, two of the eight cells of the 2x2x2 results table would be unobserved (sometimes called a ‘structural zero’), the negative predictive value of the OR rule could not be estimated directly, and models that implicitly or explicitly contain parameters that correspond to related statistics would not be identifiable.

One solution is to impose constraints over some of the parameters governing the multinomial model. Two such ‘capture-recapture’ estimators have been termed ‘homogeneous dependence’ ($\text {P}(X_1=1|X_2=1)/\text {P}(X_1=1)$ is constant irrespective of *T*) and ‘homogeneous odds ratio’ (the odds ratio of two index test results is constant irrespective of *T*) [101]. These constraints are extended to ‘homogeneous relative risk’, ‘homogeneous gamma’ and ‘homogeneous Kappa coefficient’, all similarly defined in terms of other common measures of association [79]. Direct estimators for the rate ratio have also been considered [102].

### Meta-analysis

Methods for performing meta-analysis of diagnostic accuracy data in the case of single index tests are widely used [103]. Less attention has been paid to the meta-analysis of diagnostic test sequences, except in the scenario in which a test sequence can be regarded as comprising a single index test in its own right, in which case standard methods can be used, provided all individuals in all contributing studies have undergone each of the index tests that constitute the test sequence.

An exception is the pair of papers by Novielli et al., who performed a meta-analysis of two index tests (D-dimer and Wells score) performed in combination [104, 105]. In some of the contributing studies, a conditional testing format occurred, by which test implementation depended on a previous test result, and so index test results for some participants were incomplete. The authors implemented a meta-analytic model that allows for a variety of data types, reflecting different study designs, which contain parameters that allow for conditional dependence between test results as well as dependence between sensitivity and specificity for each index test [104]. They also showed that cost-effectiveness considerations were affected by the strength of the dependence between index test results, which suggests that allowing for this dependence is an important consideration in future evidence syntheses of similar types of test sequence [105]. These methods remain under-used and may be easier to implement in individual patient data meta-analysis than in aggregate-level data meta-analysis [106], although recent methodological developments have included a proposed network meta-analytic model for multiple diagnostic tests [107].

#### Health-economic models

Implications for health-economic models, which often rely on estimates of test performance from meta-analysis as input parameters to the model, are discussed in a recent review [5]. A common challenge, as noted in the section Estimating conditional dependence between index tests performed in sequence, and conditional testing, is obtaining an estimate of the conditional correlation parameters (or the corresponding covariance parameters): these are unavailable if the meta-analysis relies on data from index tests that have been performed separately in different studies, and may not always be reported even in primary studies where each index test of the sequence is evaluated in the same population. In these situations, it is recommended to check the extent to which the overall performance of the test sequence is affected by the values of the covariance parameters, inside the range within which they are constrained to lie, as we illustrate in the subsequent example.

### Example: repeat faecal immunochemical testing

The FIT (Faecal Immunochemical Test) detects the degradation products of human haemoglobin in faeces (faecal occult blood). Patients with a positive FIT result detected during the investigations of symptoms or during asymptomatic screening are referred for further investigation by colonoscopy. The Oxford FIT study is a retrospective cohort study included consecutive FIT samples sent to Oxford University Hospitals Trust clinical biochemistry laboratory from primary care for symptomatic adults (age $\ge$18 years) between March 2017 and March 2020 [108]. The data are shown in Table 2. Here, a positive index test result is defined as a FIT result exceeding a threshold of 10$\mu$g Hb/g faeces. Reference test results for a diagnosis of colorectal cancer were obtained from linked hospital data reflecting a composite of laboratory, endoscopy and histopathology records.

**Table 2:** Repeat faecal immunochemical testing data

First FIT result	Second FIT result	Reference standard result	Number of participants
$+$	$+$	$+$	8
$+$	$+$	−	63
$+$	−	$+$	1
$+$	−	−	66
−	$+$	$+$	0
−	$+$	−	48
−	−	$+$	1
−	−	−	1122

#### Analytic strategy

We can use the results of the previous sections and Table 1 to guide the analytic strategy for this dataset. Since we will often want to determine the diagnostic accuracy for a sequence of tests, but will only be in possession of data from a single test, we start by showing how the accuracy of a sequence of tests could be estimated by making certain assumptions about the interdependence between them, acting initially as though we only have reference standard data, and index test data from the first time point (Estimating conditional dependence between index tests performed in sequence, and conditional testing section). We will then use the full dataset, which also uses index test data from the second time point, to illustrate the performance of the OR rule for combining two index test results (Combining two index tests section). In this scenario, the reference standard is likely to misclassify some individuals, making this dataset suitable for analysis using latent variable methods to adjust for imperfect reference standard bias (Analysing test sequences in conjunction with an imperfect or incomplete reference standard section), which allows us to assess the impact of this compared with the unadjusted results.

#### Estimating the accuracy of a sequence of tests using data from a single test

For illustration we use data from only the first, third and fourth columns of Table 2 in this section. Based on the first FIT, 9 out of 10 colorectal cancer cases and 129 out of 1299 individuals without cancer received a positive FIT result, giving estimates (95% confidence interval, CI) of 0.900 (0.555 to 0.997) for the sensitivity and 0.901 (0.883 to 0.916) for the specificity.

To estimate the diagnostic performance of a repeat FIT in combination with the first FIT, the simplest assumption is that the tests are conditionally independent, given true disease status. More plausible estimates could be obtained by assuming the tests are positively correlated. In the absence of data on the correlation, we think it sensible to try a range of correlations corresponding to low, moderate and high levels of dependence. Assuming that repeated measurements are conditionally independent ($\rho _{+} = \rho _{-} = 0$), the sensitivity and specificity of a sequence of two FITs using the OR rule (equation 1) are:

$$\begin{aligned} Se_{X_{1} \vee X_{2}}{} & {} = 0.900 + (1 - 0.900) \times 0.900 - 0 = 0.990 \\ Sp_{X_{1} \vee X_{2}}{} & {} = 0.901 \times 0.901 + 0 = 0.811 \end{aligned}$$

with 95% CIs 0.643 to 0.995 for the sensitivity and 0.789 to 0.832 for the specificity, calculated using the Wilson-score interval method [109]. Table 3 shows the estimated sensitivity and specificity under independence, and three scenarios relating to low, moderate and high levels of positive correlation between tests.

**Table 3:** Estimates of sensitivity and specificity of the OR decision rule for a sequence of two tests under four different scenarios corresponding to no, low, moderate and high correlation between tests

Assumed correlation	Sensitivity $\varvec{(Se}_{\varvec{X}_{\varvec{1}} \varvec{\vee } \varvec{X}_{\varvec{2}}}\varvec{)}$	Specificity ($\varvec{Sp}_{\varvec{X}_{\varvec{1}} \varvec{\vee } \varvec{X}_{\varvec{2}}}$)
$\rho _{+} = \rho _{-} = 0$	0.990	0.811
$\rho _{+} = \rho _{-} = 0.25$	0.968	0.834
$\rho _{+} = \rho _{-} = 0.50$	0.945	0.856
$\rho _{+} = \rho _{-} = 0.75$	0.922	0.878

This illustrates the results presented in the section Combining two index tests and the Appendix: for the OR rule, the dependence between the tests induces a decrease in the sensitivity and an increase in the specificity of the test sequence, compared to the case where independence is assumed.

#### Direct estimation from a sequence of test results

Having access to repeat FIT results in a subset of patients allows us to estimate the sensitivity and specificity for different decision rules directly, and also allows us to quantify the dependency between repeat tests. Of the nine reference standard-positive cases that had a positive FIT on the first test, eight were again positive on the second test. The one false negative on the first test was also negative on the second test (Table 2).

The estimated sensitivity of the OR rule is therefore $9/10 = 0.900$ with $95\%$ confidence interval 0.596 to 0.982. The observed correlation between these repeat FIT in patients with cancer was estimated to be $\rho _{+} = 0.667$. Of the 1299 patients without cancer, 1122 were negative on both tests (Table 2) so the specificity of the OR rule is estimated to be $1122/1299 = 0.864$, with $95\%$ confidence interval 0.844 to 0.881. The correlation between repeat FIT in the non-cancer patients was estimated to be $\rho _{-} = 0.479$, lower than the correlation between repeat tests in patients with cancer.

#### Choosing the optimal strategy

As discussed in the section Combining two index tests, choosing the optimal strategy will often involve a trade-off between sensitivity and specificity, and careful consideration of both the prevalence of the condition and the consequences of the false-positive and false-negative decisions. In this context, the desire to avoid false negative diagnoses suggests that OR rule is likely to be more appropriate than the AND rule, as the latter cannot increase the sensitivity compared to using a single test.

Repeating the FIT and using an OR rule could improve the sensitivity over and above a single FIT but it would be at the expense of the specificity. The empirical analysis suggests that the repeat strategy would increase the number of false positives by 48/1299 or $\sim 37$ per 1000 (3.7%). For sensitivity, the approach based on single test data and assuming a moderate correlation ($\rho _{+}=0.5$) appeared to indicate that sensitivity could hypothetically be increased from 0.900 for a single test to 0.945 using a repeat strategy. This corresponds to one fewer false negative per 2250 patients investigated with FIT (assuming a similar prevalence). The empirical analysis did not show any change in sensitivity, but due to the low number of cases in this data set ($n = 10$) there is considerable uncertainty around these estimates. Similar to the challenges of estimating single test diagnostic accuracy in low prevalence settings [110], obtaining precise estimates of the sensitivity of sequential test strategies can be challenging empirically. Further, the hypothetical approach assumes no change in disease status or in test performance over time, and this assumption may not always hold.

#### Adjusting for imperfect reference standard bias

In this scenario we have complete but imperfect reference standard verification, and so can use Bayesian latent variable methods as described in the section Analysing test sequences in conjunction with an imperfect or incomplete reference standard, where the latent variable represents the true colorectal cancer status *T*. We follow the parameterisation adopted by other authors [26, 28, 88].

Generally, the latent variable model might include a disease prevalence parameter, sensitivity and specificity parameters for both index tests and the reference test, and two conditional covariance parameters for each pair of index and/or reference tests. As there are only eight possible combinations of index/reference test results, the full parameterisation would make the model non-identifiable.

We therefore assume that the two uses of the index test share the same sensitivity parameter and share the same specificity parameter, since this is the same test used twice rather than two different index tests. For the same reason, we allow two conditional covariance parameters (conditional on disease-positive and disease-negative cases, respectively) to model the association between the two index test results, but assume that the reference test result is independent of the index test result, given true disease status.

As reference standard records of a cancer diagnosis are unlikely to be false positives, we assume that the reference standard has 100% specificity and high (but sub-100%) sensitivity. The full specification of the multinomial model, and prior distributions for the reference standard sensitivity and the other parameters, are described in full in Supplementary Material.

**Fig. 4:**
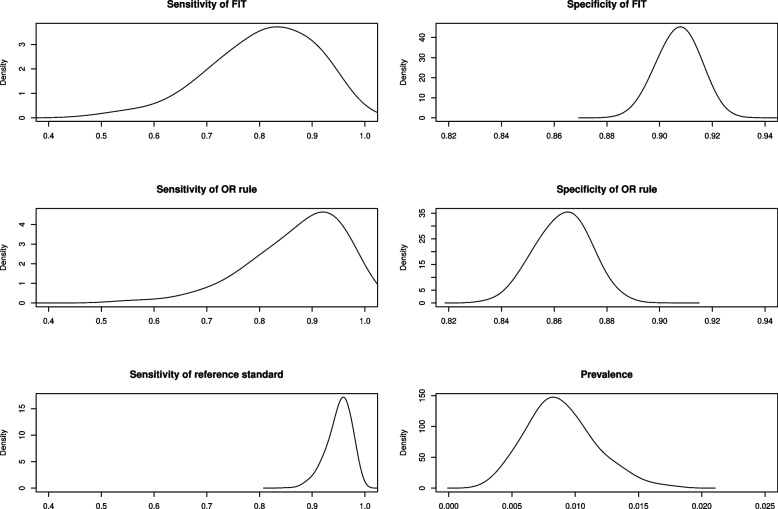
Posterior distributions for selected parameters, from latent variable model adjusting for imperfect reference standard bias

Table 4 and Fig. 4 shows parameter estimates from the latent variable model. Compared to the results from the previous sections, in this example adjusting for imperfect reference standard bias in conjunction with modelling the conditional dependence between index test results tends to reduce the estimated sensitivity of the diagnostic test, while having a lesser effect on its estimated specificity. These results are illustrative and are influenced by the reference standard priors.

**Table 4:** Parameter estimates from latent variable model adjusting for imperfect reference standard bias

Parameter	Median of posterior distribution (95% credible interval)
Sensitivity of FIT	0.817 (0.565, 0.959)
Specificity of FIT	0.907 (0.893, 0.920)
Sensitivity of OR rule	0.890 (0.658, 0.987)
Specificity of OR rule	0.864 (0.844, 0.882)
Sensitivity of reference standard	0.955 (0.895, 0.986)
$\rho _{+}$	0.562 (0.071, 0.925)
$\rho _{-}$	0.477 (0.395, 0.560)
Prevalence $(\pi )$	0.00860 (0.00431, 0.0148)

## Discussion

This paper has presented a methodological review of statistical methods that can be used to analyse data from studies of the accuracy of diagnostic tests performed in sequence. Our aim in writing this paper was to guide analysts towards the class of methods that are likely to be of most benefit when deciding on a suitable analytic strategy for data of this type. We have classified methods by the purpose for which they are most likely to be used, resulting in the five groupings of methods we have described.

Methodological research in this field lacks a common vocabulary, which made the literature search challenging and a fully systematic search implausible. Even the term ‘sequential testing’ is easily confused with the separate practice of performing repeated statistical hypothesis testing such as might be used in adaptive clinical trials. In writing our review we have tried to draw attention to terminology that has been used interchangeably, in the hope that this will help other researchers to navigate the relevant research literature, and propose the term ‘sequential diagnostic testing’ as the most appropriate descriptor.

Another characteristic of work in this field is the tendency for analytical requirements to span different methodological areas. For example, studies from screening programmes might require an assessment of the diagnostic accuracy, number and order of several screening tests in combination, while simultaneously making allowance for a reference test that is only partially performed and may itself be imperfect. The frequency of designs of this type may explain why many papers related to sequential diagnostic tests also seek to address reference standard classification issues, even though the latter may be regarded as a separate methodological area in its own right. Understandably, many methodological developments have been made with the idiosyncrasies of a particular study application in mind, which presents a challenge when trying to identify commonalities between methods and in providing general methodological recommendations. For this reasons, the groupings of methods that we have identified should not be interpreted in isolation, but rather in conjunction with one another. Likewise, our review complements other methodological reviews in related areas [74–76, 111].

Many of the methods identified aim to address what might be described as a missing data problem - whether because of conditional index testing, partial verification, an imperfect reference standard, or a combination of these. Some of the methods in common use, such as latent variable methods, reflect this. It should be emphasised though that there is ‘no free lunch’ when implementing these methods: in diagnostic studies, there is often a low limit to the number of parameters that can be estimated without making simplifying assumptions, no matter how complex the method appears to be. For this reason, methods that aim to synthesise information from multiple studies are likely to be of value in future research.

Our case study highlights the importance of allowing for conditional dependence between diagnostic tests when they are used as part of a sequence. This issue is of particular importance when testing diagnostic pathways for which there is limited data about the relevant dependence parameters [4, 5, 22]. Analysts should therefore be aware that model outputs should provide this information, or that data should be presented in a suitable way to calculate them directly. In simpler examples, this might require no more than to present multidimensional contingency tables that cross-tabulate index test and reference test results. This requirement is likely to grow in importance as meta-analytic methods develop.

Our paper has some limitations. As we did not intend the review to be fully systematic, it is possible that some relevant papers were not captured by our search strategy. The overlap between methodological concepts means that it has not been possible to provide a tool, such as a flowchart, which might more clearly signpost the preferred method for a given design, although the supplementary table may be used as a guide to the key methodological issues to be considered. An appropriate choice of method is likely to be highly context-dependent, and our paper may act as a reference to help locate where the most useful previous publications are likely to be found.

Except for the case study described, the review has not provided details of computational routines, which are burdensome for many of the more advanced methods described, or of software implementation. We found that, despite their complexity, only a small number of the suggested methods have user-friendly software routines available, and we suggest that the provision of software should also be a priority for improving access to existing methods.

## Conclusion

Our review has described a variety of methodological approaches for sequential diagnostic testing. We have outlined five themes that link these methods, depending on the objectives at hand, and suggest that these be used as a way to guide future methodological development in this field.

### Supplementary Information

Supplementary Material 1.

Supplementary Material 2.

## Data Availability

All data required to recreate the results in this paper are included within the main body of the manuscript.

## Appendix

### Notation and additional formulae

In a population with disease prevalence $\pi$, we write the true disease status $T_i$ for individual *i* as $T_i=1$ for disease positives, and $T_i=0$ for disease negatives. Let $X_{1i}$ denote the outcome of the first test for individual *i*, such as in Fig. 1, and $X_{2i}$ denote the outcome of the second test, although for simplicity we suppress the *i* subscripts below.

Following the notation of Thompson [41], we define $X_1 \wedge X_2$ as ($X_{1}=1$ and $X_{2}=1$), and $X_1 \vee X_2$ as ($X_{1}=1$ or $X_{2}=1$). Therefore, $X_1 \wedge X_2$ produces a positive diagnosis if and only if $X_{1}$ and $X_{2}$ are both positive, while $X_1 \vee X_2$ produces a positive diagnosis if at least one of $X_{1}$ and $X_{2}$ is positive. We use $\text {Se}(\cdot )$ and $\text {Sp}(\cdot )$ for the sensitivity and specificity, respectively, for a test or combination of tests, and $\text {PPV}(\cdot )$ and $\text {NPV}(\cdot )$ for the positive and negative predictive values. The symbol $\lnot$ represents negation.

The conditional correlations between two index test results given positive and negative disease status, are respectively defined as

$$\begin{aligned} \rho _{+}=\text {Corr}(X_1,X_2|T=1) \end{aligned}$$

and

$$\begin{aligned} \rho _{-} = \text {Corr}(X_1,X_2|T=0). \end{aligned}$$

The OR rule has sensitivity

$$\begin{aligned} \text {Se}(X_1 \vee X_2){} & {} = \text {P}(\lnot (X_1=0,X_2=0)|T=1)\\{} & {} = 1 - \text {P}((X_1 = 0, X_2 = 0)|T = 1)\\{} & {} = 1 - \text {P}(X_2=0|X_1=0,T=1)(1-\text {Se}(X_1)) \end{aligned}$$

and specificity

$$\begin{aligned} \text {Sp}(X_1 \vee X_2){} & {} = \text {P}(X_1=0,X_2=0|T=0)\\{} & {} = \text {P}(X_2=0|X_1=0,T=0)\text {Sp}(X_1) \end{aligned}$$

while the AND rule has sensitivity

$$\begin{aligned} \text {Se}(X_1 \wedge X_2){} & {} = \text {P}(X_1=1,X_2=1|T=1)\\{} & {} = \text {P}(X_2=1|X_1=1,T=1)\text {Se}(X_1) \end{aligned}$$

and specificity

$$\begin{aligned} \text {Sp}(X_1 \wedge X_2){} & {} = \text {P}(\lnot (X_1=1,X_2=1)|T=0)\\{} & {} = 1 - \text {P}(X_2=1|X_1=1,T=0)(1-\text {Sp}(X_1)). \end{aligned}$$

It can further be shown that:

$$\begin{aligned} \text {Se}(X_1 \vee X_2){} & {} \ge \max \{\text {Se}(X_1),\text {Se}(X_2)\}\\ \text {Sp}(X_1 \vee X_2){} & {} \le \min \{\text {Sp}(X_1),\text {Sp}(X_2)\}\\ \text {Se}(X_1 \wedge X_2){} & {} \le \min \{\text {Se}(X_1),\text {Se}(X_2)\}\\ \text {Sp}(X_1 \wedge X_2){} & {} \ge \max \{\text {Sp}(X_1),\text {Sp}(X_2)\}. \end{aligned}$$

The expressions for $\text {Se}(X_1 \vee X_2)$ and $\text {Sp}(X_1 \vee X_2)$ can be written in terms of the phi coefficient for measuring association [29]:

1 $$\begin{aligned} \text {Se}(X_1 \vee X_2) = \text {Se}(X_1) + (1-\text {Se}(X_1))\text {Se}(X_2) - \tau _{+} \end{aligned}$$

where

$$\begin{aligned} \tau _{+}=\rho _{+}[\text {Se}(X_1)\text {Se}(X_2)(1-\text {Se}(X_1))(1-\text {Se}(X_2))]^{0.5}, \end{aligned}$$

and

$$\begin{aligned} \text {Sp}(X_1 \vee X_2) = \text {Sp}(X_1)\text {Sp}(X_2) + \tau _{-} \end{aligned}$$

where

$$\begin{aligned} \tau _{-}=\rho _{+}[\text {Sp}(X_1)\text {Sp}(X_2)(1-\text {Sp}(X_1))(1-\text {Sp}(X_2))]^{0.5}. \end{aligned}$$

Here, $\rho _{+}$ and $\rho _{-}$ (as defined previously) are equivalent to the measure sometimes known as the phi coefficient for measuring association between binary variables, calculated among disease positive and disease negative cases respectively [25].

The likelihood function referred to in the Results “Estimating conditional dependence between index tests performed in sequence, and conditional testing” section, for modelling test sequence data as a realisation of a multinomial distribution with prevalence $\pi$, in the case of two binary tests, is

2 $$\begin{aligned} L=\prod \limits _{i,j=0}^1\{\pi \text {P}(X_1=i,X_2=j|T=1) + (1-\pi ) \text {P}(X_1=i,X_2=j|T=0) \}^{x_{ij}}, \end{aligned}$$

where the $x_{ij}$ are the observed number of individuals for each possible combination of index test results [58].

The reparameterisation of the multinomial model referred to in the Results “Analysing test sequences in conjunction with an imperfect or incomplete reference standard” section uses terms of the form

$$\begin{aligned} \text {P}(X_1=1,X_2=1|T=1) = \text {P}(X_1=1|T=1)\text {P}(X_2=1|T=1)+c_1, \end{aligned}$$

similarly to equation (1). Here $c_1=\text {Cov}(X_1,X_2|T=1)$ is a conditional covariance term, given the true disease status is positive, and that the joint probability terms for other possible index test result pairs can be expressed similarly in terms of either $c_1$ or $c_0=\text {Cov}(X_1,X_2|T=0)$.

## Abbreviations

FIT: Faecal immunochemical test
NPV: Negative predictive value
PPV: Positive predictive value
ROC: Receiver operating characteristic
