# Supplementary material for: A review of methods for the analysis of diagnostic tests performed in sequence
Source: Diagn Progn Res. 2024 Sep 3;8:8. doi: 10.1186/s41512-024-00175-3 (PMC11370044; doi:10.1186/s41512-024-00175-3)
Supplement: Supplementary file 1 — Supplementary Material 1. [file 41512_2024_175_MOESM1_ESM.docx]

| How many index tests are being evaluated? | *If only one, this is outside the scope of this review*  *If two, see Results Section 1.1*  *If more than two, see Results Section 1.2* |
| --- | --- |
| Do available data take the form of summaries from existing studies, rather than primary data? | *If yes, see Results Section 5* |
| Are all index test results available for all participants? | *If yes, see Results Section 1*  *If no, see Results Section 2* |
| Is a new index test being added to an existing test sequence? | *If yes, see Results Section 1.1* |
| Has a method for combining the results of all index tests in the sequence already been established? | *If no, see Results Section 1.2* |
| Is an aim to estimate or model the conditional correlation between index tests? | *If yes, see Results Section 2* |
| Are reference test results available for all participants? | *If no, see Results Section 4* |
| Can the reference test be considered as a gold standard? |  |
| Are there additional methodological considerations that are specific to screening or monitoring programmes? | *If yes, see Results Section 3* |

*Supplementary Table. Some methodological considerations when analysing test sequences of binary diagnostic index tests, with indicative sections of the paper in which relevant information appears. Sections are not mutually exclusive and for many test sequences, more than one section may be relevant.*
