# Supplementary material for: A review of methods for the analysis of diagnostic tests performed in sequence
Source: Diagn Progn Res. 2024 Sep 3;8:8. doi: 10.1186/s41512-024-00175-3 (PMC11370044; doi:10.1186/s41512-024-00175-3)
Supplement: Supplementary file 2 — Supplementary Material 2. [file 41512_2024_175_MOESM2_ESM.docx]

**Supplementary Material – Further details of latent variable model**

Notation:

$X_{1}$: first index (FIT) test result (1=positive, 0=negative)

$X_{2}$: second index (FIT) test result (1=positive, 0=negative)

$X_{3}$: reference test result (1=positive, 0=negative)

$T$: true colorectal cancer status (1=positive, 0=negative)

$\pi$: true colorectal cancer prevalence

$Se(\cdot)$: sensitivity of a test, abbreviated here as $S_{1},S_{2},S_{3}$ for tests $X_{1},X_{2},X_{3}$ respectively

$Sp(\cdot)$: specificity of a test, abbreviated here as $C_{1},C_{2},C_{3}$ for tests $X_{1},X_{2},X_{3}$ respectively

${Cov_{12}}^{+}$: covariance between $X_{1}$ and $X_{2}$, conditional on $T=1$

${Cov_{12}}^{-}$: covariance between $X_{1}$ and $X_{2}$, conditional on $T=1$

The latent variable approach models the observed test results as a realisation of a multinomial distribution, such that $P\left( X_{1}=x_{1},X_{2}=x_{2},X_{3}=x_{3} \right)$ is equal to

$$P\left( X_{1}=x_{1},X_{2}=x_{2},X_{3}=x_{3}|T=1 \right)P(T=1)+ P\left( X_{1}=x_{1},X_{2}=x_{2},X_{3}=x_{3}|T=0 \right)P(T=0)$$

Following Menten et al (2008), in the general model allowing for conditional dependence between$X_{1}$ and $X_{2}$ (conditional on $T$), but conditional independence between $X_{3}$ and the index tests, this can be rewritten as

$\pi\left( {S_{1}}^{x_{1}}{S_{2}}^{x_{2}}{{(1-S}_{1})}^{\left( 1-x_{1} \right)}{{(1-S}_{2})}^{\left( 1-x_{2} \right)}+\left( -1 \right)^{x_{1}-x_{2}}{Cov_{12}}^{+} \right){S_{3}}^{x_{3}}{(1-S_{3})}^{\left( 1-x_{3} \right)}$+

$$(1-\pi)\left( {C_{1}}^{{(1-x}_{1})}{C_{2}}^{(1-x_{2})}{{(1-C}_{1})}^{x_{1}}{{(1-C}_{2})}^{x_{2}}+\left( -1 \right)^{x_{1}-x_{2}}{Cov_{12}}^{-} \right){C_{3}}^{(1-x_{3})}{(1-C_{3})}^{x_{3}}$$

Further assuming that $C_{3}=1$, i.e. that the nature of the reference test does not allow the possibility of false positive cancer diagnoses, reduces to zero the contribution towards the likelihood function of the second line of the above expression when the true cancer status is negative.

As the index (FIT) test is the same test conducted on two occasions, we assume $S_{1}=S_{2}$ and $C_{1}=C_{2}$. Then the conditional correlation parameters are $\rho_{+}={Cov_{12}}^{+}/(S_{1}\left( 1-S_{1} \right))$and $\rho_{-}={Cov_{12}}^{-}/(C_{1}\left( 1-C_{1} \right))$. Estimates of the diagnostic performance of the OR rule can be calculated using the expressions for $Se(X_{1}\vee X_{2}$) and $Sp(X_{1}\vee X_{2}$) provided in the Appendix.

Priors for $\pi$, $S_{1}$ and $S_{2}$ are each set as $\mathrm{Beta}\left( 1,1 \right)$, i.e. a uniform distribution on $[0,1]$. The prior for $S_{3}$ is informative and guided by the assumed likely sensitivity of the reference standard. We hypothesise (as a best estimate) that 1 in 20 true colorectal cancer cases may go unrecorded by the reference standard, and this figure is unlikely to be more than 1 in 10 or less than 1 in 100. As such, we determined the parameters that correspond to a Beta distribution with mean 0.95 and with 95% of the density lying between 0.90 and 0.99. This implies a $\mathrm{Beta}\left( 72.253,3.750 \right)$ prior for $S_{3}$.

The positive and negative covariance parameters are bounded above by $S_{1}-{S_{1}}^{2}$ and $C_{1}-{C_{1}}^{2}$ respectively (Dendukuri & Joseph (2001)), to ensure that each element of the multinomial distribution is a valid probability, and so $U(0,S_{1}-{S_{1}}^{2}$) and $U(0,C_{1}-{C_{1}}^{2}$) priors are used for these parameters.

Model fitting was carried out using WinBUGS14 via the R2WinBUGS package (Sturtz et al (2005)) in R version 4.2.1 (R Core Team 2022), with a burn-in of 1000 and 10000 subsequent iterations thinned by a factor of 10. Diagnostic checks were consistent with adequate convergence.

References

Dendukuri N, Joseph L. Bayesian approaches to modeling the conditional dependence between multiple diagnostic tests. Biometrics. 2001;57:158–167.

Menten J, Boelaert M, Lesaffre E. Bayesian latent class models with conditionally dependent diagnostic tests: a case study. Stat Med. 2008;27:4469–4488.

R Core Team. R: A Language and Environment for Statistical Computing. R Foundation for Statistical Computing: Vienna, Austria. 2005; https://www.R-project.org.

Sturtz S, Ligges U, Gelman A. R2WinBUGS: A Package for Running WinBUGS from R. J Stat Softw. 2005;12:1-16.

**Model**

model{

n<-sum(freq[1:8])

# The multinomial likelihood has 8 cells for combinations of the two index test and the reference test results

freq[1:8]~dmulti(p[1:8],n)

p[1]<-prev*(set*set+covp)*ser+0

p[2]<-prev*(set*set+covp)*(1-ser)+(1-prev)*((1-spt)*(1-spt)+covn)*1

p[3]<-prev*(set*(1-set)-covp)*ser+0

p[4]<-prev*(set*(1-set)-covp)*(1-ser)+(1-prev)*(spt*(1-spt)-covn)*1

p[5]<-prev*(set*(1-set)-covp)*ser+0

p[6]<-prev*(set*(1-set)-covp)*(1-ser)+(1-prev)*(spt*(1-spt)-covn)*1

p[7]<-prev*((1-set)*(1-set)+covp)*ser+0

p[8]<-prev*((1-set)*(1-set)+covp)*(1-ser)+(1-prev)*(spt*spt+covn)*1

#Priors

prev~dbeta(1,1)

set~dbeta(1,1)

spt~dbeta(1,1)

ser~dbeta(72.253,3.750)

ubp<-set-pow(set,2)

ubn<-spt-pow(spt,2)

covp~dunif(0,ubp)

covn~dunif(0,ubn)

#Correlations

rhop<-covp/sqrt(set*(1-set)*set*(1-set))

rhon<-covn/sqrt(spt*(1-spt)*spt*(1-spt))

#Predictive values of index test, calculated from sens, spec and prevalence

ppvt<-(set*prev)/((set*prev)+(1-spt)*(1-prev))

npvt<-(spt*(1-prev))/(spt*(1-prev)+(1-set)*prev)

# Prediction

freqpred[1:8]~dmulti(p[1:8],n)

#Diagnostic performance of the OR rule

taup<-rhop*set*(1-set)

seor<-set+(1-set)*set-taup

taun<-rhon*spt*(1-spt)

spor<-spt*spt+taun

}

**Initial values**

list(prev=0.5,set=0.95,spt=0.95,ser=0.95,covp=0.03,covn=0.03,freqpred=c(0,0,0,0,0,0,0,1309))

**Data**

#Data are entered in the object freq in this order:

#+ index test1, + index test2, + reference test

#+ index test1, + index test2, - reference test

#+ index test1, - index test2, + reference test

#+ index test1, - index test2, - reference test

#- index test1, + index test2, + reference test

#- index test1, + index test2, - reference test

#- index test1, - index test2, + reference test

#- index test1, - index test2, - reference test

list(freq=c(8,63,1,66,0,48,1,1122))
